# Harnessing machine learning to predict and prevent proximal junctional kyphosis and failure in adult spinal deformity surgery: A systematic review

**DOI:** 10.1016/j.bas.2025.104273

**Published:** 2025-05-05

**Authors:** Paolo Brigato, Gianluca Vadalà, Sergio De Salvatore, Leonardo Oggiano, Giuseppe Francesco Papalia, Fabrizio Russo, Rocco Papalia, Pier Francesco Costici, Vincenzo Denaro

**Affiliations:** a Research Unit of Orthopaedic and Trauma Surgery, Department of Medicine and Surgery, Università Campus Bio-Medico di Roma, Via Alvaro del Portillo, Roma, 21 - 00128, Italy; b Fondazione Campus Bio-Medico di Roma, Via Alvaro del Portillo 200, Roma 00128, Italy; c Orthopedic Unit, Department of Surgery, Bambino Gesù Children's Hospital, Rome, Italy

**Keywords:** Proximal junctional kyphosis, Proximal junctional failure, Adult spinal deformity, ASD, Predictive modelling, Machine learning, artificial intelligence, AI

## Abstract

**Introduction:**

Adult spinal deformity (ASD) surgery involves high costs and risks, with Proximal Junctional Kyphosis (PJK) and Proximal Junctional Failure (PJF) being major concerns. Artificial intelligence (AI) and machine learning (ML) offer potential in predicting and preventing these complications. This review examines the role of AI in predicting PJK/PJF, its effectiveness, and future research needs.

**Research question:**

Can AI-based models accurately predict PJK/PJF after ASD surgery, and what factors affect their performance?

**Material and methods:**

A systematic review was conducted following PRISMA guidelines, analyzing Medline, Scopus, Embase, and Cochrane Library databases up to December 2024. Keywords included “Adult Spinal Deformity,” “PJK,” “PJF,” “AI,” and “ML.” Data extracted included study characteristics, patient demographics, surgical details, AI model parameters, and performance metrics. Bias risk was assessed using the MINORS score.

**Results:**

Among 164 studies, 7 met inclusion criteria (n = 2179 patients). Mean age was 63.2 ± 3.7 years, BMI 26.1 ± 2.4 kg/m^2^, and fusion levels 9.82 ± 1.8. PJK/PJF occurred in 41.1 %. AI models (Random Forest, supervised learning) had accuracy from 72.5 % to 100 % (AUC up to 1.0). Key predictors included age, BMD, spinal alignment, and implant type.

**Discussion and conclusions:**

AI and ML models show promise in predicting PJK/PJF after ASD surgery. However, larger multicenter studies with standardized definitions, BMD assessments, and preoperative MRI integration are needed for broader clinical application and validation.

## Highlights

• Machine learning enhances early risk identification for PJK after adult spinal deformity surgery.
• RCTs should validate ML predictions by integrating standardized PJK/PJF definitions, preoperative MRI, and key radiographic parameter.
• Accurate BMD assessment, UIV selection, and prophylactic measures are crucial for improving predictive models.

## 1 Introduction

Adult spinal deformity (ASD) refers to a diverse group of medical disorders characterized by alterations in the coronal and axial profiles, which typically arise in older adulthood (de Kleuver et al., 2021; Diebo et al., 2019). As the general population ages, the prevalence of ASD is expected to increase, with over 60 million older adults in the United States projected to have some form of spinal deformity by 2050 (Ames et al., 2016). The development of ASD is typically associated with the onset of various types of pain symptoms, such as back pain, radiculopathy, and postural instability, which result in a significant reduction in quality of life (Kanter et al., 2014). Compared to conservative treatment, surgical intervention improves patient-reported outcomes but also incurs significant healthcare-related financial costs (Alvarado et al., 2021; Arutyunyan et al., 2018). Moreover, the high risk of medical and mechanical complications during the perioperative period and in the long term further complicates the therapeutic decision-making process (Smith et al., 2019; Passias et al., 2023a; Soroceanu et al., 2016).

Among the most common mechanical complications of surgical treatment for ASD are those referred to under the umbrella term of proximal adjacent segment pathology (ASP), including Proximal Junctional Kyphosis (PJK) and Proximal Junctional Failure (PJF) (Nguyen et al., 2016). Although its definition remains inconsistent, PJK is generally characterized by a progressive increase in the Proximal Junctional Angle (PJA) between the uppermost instrumented vertebra (UIV) and the vertebra two levels above (UIV + 2), with an incidence ranging from 20 % to 40 % of patients (Kim et al., 2013; Zhao et al., 2021; Cerpa et al., 2020). If left untreated, PJK may advance to symptomatic PJF, which may require revision surgery in cases of impaired structural integrity or neurological deficits (Nguyen et al., 2016; Yagi et al., 2023; Hart et al., 2013).

Over the years, numerous efforts have been made to identify risk factors and develop preventive strategies for complications by creating models that combine demographic, radiographic, and surgical data and advanced analytical techniques, such as regressions (Zhao et al., 2018, 2021, 2023; Tian et al., 2024; Alvarez et al., 2022; Kuo et al., 2023). Despite these advancements, a definitive consensus on accurately predicting ASP development remains elusive, as the complexity of its pathophysiology and numerous variables pose significant challenges in creating a universally reliable predictive framework, mainly because methods often rely on averages without accounting for individual variations or generate odds/hazard ratios for each variable (Scheer et al., 2015, 2016).

In recent years, the progressive integration of artificial intelligence (AI) in medicine has revolutionized the approach to diagnosis and therapeutic decision-making, thanks to its ability to analyze and learn from large amounts of data (Hamet and Tremblay, 2017). In the field of spinal surgery, machine learning (ML) and deep learning (DL) models have brought tangible benefits, particularly in screening, spinal parameter calculation, and predicting the development of complications, especially after surgical interventions (Hornung et al., 2022; Zhang et al., 2023; Scheer and Ames, 2024).

The potential role of AI in predicting the development of ASP could be relevant, enabling more accurate identification of patients at higher risk for developing PJK and PJF, thus enhancing clinical decision-making and precision medicine.

This article aims to review the existing literature on the role of AI in predicting and preventing PJK and PJF following surgery for ASD, while also discussing the advantages, limitations, and prospects for improving the management of these complex conditions.

## 2 Materials and methods

### 2.1 Study selection and eligibility criteria

A systematic review was performed to explore research on the application of AI-based predictive models for forecasting the development of PJK/PJF after surgical treatment for ASD, adhering to the Preferred Reporting Items for Systematic Reviews and Meta-Analyses (PRISMA) guidelines (Haddaway et al., 2022). The inclusion and exclusion criteria were established before the commencement of the study (Table 1). The formulation of the research question was done using a PIOS approach: Population (P), Intervention (I), Outcome (O), and Study Design (S). This systematic review collected and analyzed data on patients who underwent ASD surgery (P). ML-based predictive models (I) were adopted to evaluate accuracy and/or Area Under the Curve (AUC)/Area Under the Receiver Operating Characteristics (AUROC)/Area Under The Precision-Recall Curve (AUPRC) in the prediction of PJK/PJF (O). The following study designs were included (S): Randomized Control Trials (RCT), Retrospective Cohort studies (RC), Prospective Cohort studies (PC), and comparative studies.

**Table 1:** Eligibility criteria.

INCLUSION CRITERIA	
Population	Patients with ASD older than 18 years old
Intervention	AI-based predictive models examining PJK/PJF incidence.
Outcomes	Report accuracy and/or AUC/AUROC/AUPRC in the prediction of PJK/PJF following surgery
Study design	RCTs, cohort studies, comparative studies

**EXCLUSION CRITERIA**	
Population	Patients with other deformity etiologies or outside the age range are considered.
Intervention	Studies involving non-AI predictive models, such as analytical analyses, 3D-model-based models, or finite-element methods.
Outcomes	Studies not reporting on the primary outcomes of interest. Studies with incomplete data. Studies without a clearly stated number of PJK/PJF cases.
Study design	Non-comparative studies, Reviews, editorials, case reports.

AI: Artificial Intelligence; ASD: Adult Spinal Deformity; AUC: Area Under the Curve; AUPRC: Area Under The Precision-Recall Curve; AUROC: Area Under the Receiver Operating Characteristics; PJK: Proximal Junctional Kyphosis; PJF: Proximal Junctional Failure; RCT: Randomized-Controlled Trials; 3-D: 3-Dimensional.

### 2.2 Search strategy

A thorough search of electronic databases, including Medline, Scopus, Embase, and the Cochrane Library, was performed from the inception of each database to December 2024. The search was filtered for “humans” and “English” language articles. The following search string was used: ((Adult Spinal Deformity) OR (Scoliosis) OR (Spinal Curvature)) AND ((Proximal Junctional Kyphosis) OR (PJK) OR (Proximal Junctional Failure) OR (PJF) OR (Proximal Junctional Angle) OR (PJA)) AND ((Artificial Intelligence) OR (Deep Learning) OR (Machine Learning) OR (Predictive Model)).

### 2.3 Data collection process

**Fig. 1:**
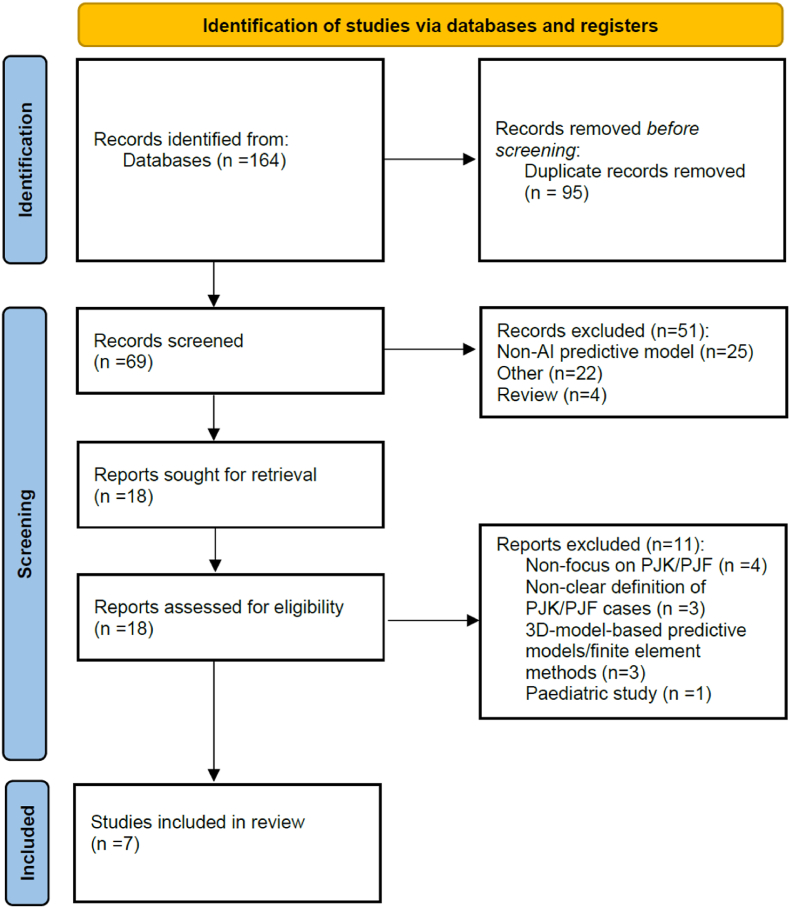
PRISMA Flow Diagram demonstrating the number of included studies.

Two independent reviewers (P.B. and S.D.S.) screened each article in two stages. Initially, article titles and abstracts were screened, followed by a full-text review of the selected articles. Any disagreements were resolved by consulting with the senior author (L.O.). The inclusion and exclusion of the reviewed articles are reported below in the PRISMA flowchart, found in Fig. 1.

### 2.4 Data items

The extracted data from each article included the first author, publication year, study type, Level of Evidence (LOE), country, journal, study objectives, inclusion and exclusion criteria, methods for radiographic parameter calculation, AI model type, training/testing ratio, definitions of PJK/PJF, and PJA measurement. Patient demographics and follow-up details, such as total patient count, case/control numbers, age, sex, and Body Mass Index (BMI), were collected. Intervention-related data, including primary or revision surgery, preoperative-Scoliosis Research Society (SRS)-Schwab coronal curve type and global balance (GB) modifier (Schwab et al., 2012), UIV and lower instrumented vertebra (LIV) levels, number of fused segments, number of 3-Column Osteotomies (3-CO), and implant type, were also recorded. Furthermore, ML algorithm performance metrics—accuracy, sensitivity, specificity, precision, recall, AUC, AUROC, and AUPRC—were analyzed.

### 2.5 Risk of bias in individual studies

The risk of bias in the included studies was assessed using the Methodological Index for Non-Randomized Studies (MINORS), which evaluates the quality of comparative studies (Table 2) (Slim et al., 2003). No RCTs were found. The MINORS tool consists of 12 items: clear study aim, inclusion of consecutive patients, prospective data collection, endpoints appropriate to the study's goal, unbiased assessment of endpoints, suitable follow-up duration, less than 5 % follow-up loss, prospective calculation of sample size, an adequate control group, contemporary comparison groups, baseline group equivalence, and proper statistical analysis. Each reviewer rated these items individually. The scoring system assigns 0 for no report, 1 for inadequate reporting, and 2 for adequate reporting, with a maximum possible global score of 20 for Non-randomized controlled trials (NRCTs). Due to its simplicity, MINORS is easily applicable to readers and researchers, and its reliability has been documented (Slim et al., 2003). Using this tool, the potential risk of bias in the selected studies was independently evaluated by two authors (P.B. and S.D.S). In cases where the reviewers disagreed, a third independent reviewer (L.O.) made the final decision.

**Table 2:** Study characteristics.

First Author and year	Type of study	Level of evidence	Country	Aim of the study	ASD Definition	Inclusion Criteria	Exclusion Criteria
Scheer et al. (2016) (Scheer et al., 2016)	Multi-center retrospective	3	United States	Develop a model to predict clinically significant PJK and PJF based on baseline demographic, radiographic, and surgical factors.	Scoliosis Cobb angle ≥20°, SVA ≥5 cm, PT ≥ 25°, and/or TK ≥ 60°.	Age >18 years Minimum of four fused vertebral levels, Complete 2-year follow-up data.	Neuromuscular etiology, Active infection or malignancy.
Yagi et al. (2018) (Yagi et al., 2018)	Multi-center retrospective	4	Japan	To build a predictive model for PJF after surgery in ASD patients, with or without including the BMD score.	Scoliosis Cobb angle ≥20°, C7SVA ≥5 cm, or PT ≥ 25°.	Age ≥50 years Minimum of five fused vertebral levels, segmental pedicle screw fixation from the UIV level to the LIV level Complete 2-year follow-up data.	Not appropriate radiographs Syndromic, neuromuscular, or other pathological condition.
Lafage et al. (2021) (Lafage et al., 2021)	Single-center retrospective	3	United States	To develop an ANN that mimics surgeon decision-making in UIV selection using preoperative data and alignment goals.	Scoliosis Cobb angle >20°, C7SVA >50 mm, PT > 25°, or PI-LL 66 > 10°.	Age ≥18 years Complete 1-year follow-up data.	Neuromuscular, congenital, paralytic, traumatic conditions.
Johnson et al. (2023) (Johnson et al., 2023)	Single-center retrospective	3	United States	To evaluate the efficacy of utilizing raw medical imaging with AI to predict radiographic PJK following ASD surgery.	NR	Minimum of five or more levels of fusion Complete 2-year follow-up.	NR
Lee et al. (2023) (Lee et al., 2023)	Multi-center retrospective	3	South Korea	To build an accurate ML model to predict the risk of PJK in patients with ASD.	SVA ≥50 mm, PT ≥ 25°, PI–LL mismatch >10°, or TK ≥ 60°.	Age ≥18 years Complete 1-year follow-up data.	Coronal deformity only, Less than 3-level fusion surgery Missed key spinopelvic parameters.
Ryu et al. (2023) (Ryu et al., 2023)	Multi-center retrospective	3	South Korea	To determine the major risk factors for UROs following corrective surgery for ASD, using ML-based prediction algorithms and game theory.	Scoliosis Cobb angle >20°, SVA >5 cm, PT > 25°, TK > 60°.	Minimum of four or more levels fused Complete 2-year follow-up.	Autoimmune, infectious, malignant, post-traumatic deformity, or other syndromic conditions; Less than four levels fused; Less than 2-year follow-up.
Tretiakov et al. (2023) (Tretiakov et al., 2023)	Multi-center retrospective	3	United States	To build a predictive model for PJK and PJF risk using new clinical, radiographic, and prophylactic factors.	Scoliosis Cobb angle ≥20°, SVA ≥50 mm, PT ≥ 25°, and/or TK > 60°.	Age ≥18 years Complete radiographic and HRQOL data preoperatively and at 6 weeks and 2 years postoperatively.	Neuromuscular etiology, Active infections or malignancy; UIV above T1 or LIV above L1

AI: Artificial Intelligence; ANN: Artificial Neural Network; ASD: Adult Spinal Deformity; BMD: Bone Mineral Density; C7SVA: C7 sagittal vertical axis; HRQOL: health-related quality-of-life; LIV: Lower Instrumented Vertebra; ML: Machine Learning; NR: Not Reported; PI-LL: pelvic incidence minus lumbar lordosis mismatch; PJK: Proximal Junctional Kyphosis, PJF: Proximal Junctional Failure; PT: pelvic tilt; SVA: sagittal vertical axis; TK: thoracic kyphosis; UIV: Upper Instrumented Vertebra; URO: Unplanned Reoperations.

## 3 Results

### 3.1 Study selection

The initial database search identified 164 records. After removing duplicates, 69 unique records remained. Titles and abstracts were screened for relevance, resulting in the exclusion of 51 records. The full texts of the remaining 18 articles were assessed for eligibility. A total of 7 studies met the inclusion criteria and were included in the study. The PRISMA flow diagram details the study selection process (Fig. 1).

### 3.2 Study characteristics

No RCTs met the inclusion criteria. All included studies were RC, either multicenter or single-center, and qualified as LOE four or higher. Seven NRCTs were identified, published between 2016 and 2023: six had a LOE of 3, and one had a LOE of 4. A total of 2179 patients were used to train, validate, and test the AI models. The studies showed acceptable homogeneity in terms of objectives, ASD definition, and inclusion and exclusion criteria. ASD was defined as a pathologic condition in patients aged> 18 years old presenting with a scoliosis coronal Cobb angle ≥20°, Sagittal Vertical Axis (SVA) ≥ 5 cm, Pelvic Tilt (PT) ≥ 25°, and/or Thoracic Kyphosis (TK) ≥60° (Passias et al., 2023b). The minimum follow-up required was one year in the studies by Lee et al. and Lafage et al. (Lee et al., 2023; Lafage et al., 2021), while the others required at least two years of follow-up imaging. Study characteristics are summarized in Table 2.

### 3.3 Risk of bias within studies

The risk of bias in the included studies was assessed using the Methodological Index for Non-Randomized Studies (MINORS) (Slim et al., 2003). On the whole, the studies exhibited a moderate risk of bias, with an average MINORS score of 16.5, indicating several methodological limitations. Notable strengths included clearly defined objectives, appropriate endpoints for the study aims, and the inclusion of consecutive patients in all studies. However, common weaknesses included the absence of prospective data collection and the lack of prospective sample size calculations, which were key factors in the heightened risk of bias. While these studies provide useful insights, the moderate risk of bias calls for careful interpretation of the results. Future research should address these methodological flaws to enhance the quality of evidence in this area. A detailed breakdown of the MINORS scores for each study can be found in Table 3.

**Table 3:** Methodological index for non-randomized studies (MINORS) score.

Author and Year	Clearly stated aim<	Inclusion of consecutive patients	Prospective data collection	Endpoints appropriate to study aim	Unbiased assessment of study endpoint	Follow-up period appropriate to study aim	<5 % lost to follow-up>	Prospective calculation of study size	Adequate control group	Contemporary groups	Baseline equivalence of groups	Adequate statistical analyses	Total score (… /24)
Scheer et al. (2016) (Scheer et al., 2016)	2	2	1	2	0	2	0	0	2	2	2	2	17/24
Yagi et al. (2018) (Yagi et al., 2018)	2	2	1	2	0	2	0	0	2	2	2	2	19/24
Lafage et al. (2021) (Lafage et al., 2021)	2	2	0	2	0	2	0	0	2	2	2	2	16/24
Johnson et al. (2023) (Johnson et al., 2023)	2	2	0	2	0	2	0	0	2	2	2	2	16/24
Lee et al. (2023) (Lee et al., 2023)	2	2	0	2	0	2	0	0	2	2	2	2	16/24
Ryu et al. (2023) (Ryu et al., 2023)	2	2	0	2	0	2	0	0	1	2	2	2	15/24
Tretiakov et al. (2023) (Tretiakov et al., 2023)	2	2	1	2	0	2	0	0	2	2	2	2	17/24

0: Not Reported; 1: Reported but inadequate; 2: Reported and adequate.

### 3.4 AI model validation and radiographical definitions

Table 4, Table 5 summarize the type of predictive AI, the data used for training, validation, and testing, as well as the definitions of PJK and PJF applied in the studies. A diverse range of AI models were employed, including C5.0 algorithms for decision tree building, Artificial Neural Networks (ANNs), Convolutional Neural Networks (CNNs), and Support Vector Machine models (SVMs). The training/testing ratio of 70:30 was consistent across the studies (Abbott, 2014), with Lafage et al. and Johnson et al. further dividing the testing samples into validation and testing subsets (Lafage et al., 2021; Johnson et al., 2023), to prevent overfitting (Kernbach and Staartjes, 2022). The studies showed mild heterogeneity in demographic, surgical, and radiographic variables. Demographically, the most commonly reported characteristics were age, sex, BMI, and prior fusion surgery. In terms of radiographic variables, several coronal and sagittal parameters were used for model training, including coronal parameters such as Cobb angles and sagittal parameters like SVA, TK, Lumbar Lordosis (LL), PT, Pelvic Incidence (PI) minus LL mismatch (PI-LL), T1 Pelvic Angle (TPA), and the SRS-Schwab adult spinal deformity classification (Schwab et al., 2012). When reported, PJA was calculated as the sagittal Cobb angle between the lower end plate of the UIV and the upper end plate of the UIV+2. Definitions of PJK varied among studies. Some defined PJK as an increase in postoperative PJA >20° compared to baseline alone (Lee et al., 2023), or in addition to a deterioration of at least one SRS-Schwab sagittal modifier (Scheer et al., 2016), while others defined it as an increase of ≥10° alone (Ryu et al., 2023; Tretiakov et al., 2023), or with an increase of at least 10° from the preoperative measurement (Lafage et al., 2021; Johnson et al., 2023). PJF definitions also varied, with most studies defining it as an increase in PJA of at least 15° that may require revision surgery (Lafage et al., 2021; Ryu et al., 2023; Tretiakov et al., 2023). Yagi et al. defined PJF as an increase of ≥20° in PJA, along with deterioration of at least one SRS-Schwab sub-category grade compared to baseline immediately postoperative, or as any type of PJK requiring revision surgery (Yagi et al., 2018).

**Table 4:** AI model training.

First Author and year	Radiographic assessment	AI models	Training/validation/testing ratio	Training/validation/testing patients	Trained demographic and surgical variables	Trained radiographic variables
cheer (2016) (Scheer et al., 2016)	Spineview®, ENSAM, Laboratory of Biomechanics, Paris, France	C5.0 algorithm decision tree + 5 different bootstrapped models (SPSS Modeler v16, IBM, Armonk, NY)	70:0:30	357:0:153	Age, gender, BMI, primary versus revision surgery, 3-CO, UIV, UIV implant type (hooks or screws), LIV, and the number of posterior vertebral levels fused.	Coronal Cobb angles, TK (T4-T12), LL, SVA, PT, PI-LL, SRS-Schwab adult spinal deformity classification.
Yagi et al. (2018) (Yagi et al., 2018)	NR	Tobuilda decision-making tree + C5.0 algorithm with 10 different bootstrapped models	70:0:30	112:0:33	Age, gender, BMD, BMI, levels fused, UIV and LIV level, primary or revision surgery.	Coronal Cobb angles, C7SVA, TK (T5-T12), LL, SS, PT, PI, PI-LL, TPA, SRS-Schwab adult spinal deformity classification.
Lafage et al. (2021) (Lafage et al., 2021)	NR	ANN model (Neural Network toolbox in MatLab 2016B (Mathworks, Inc., Natick, MA). 8 inputs, 10 hidden neurons and 1 output.	70:15:15	101:21:21	Age	Coronal and Sagittal preoperative alignment (PI-LL, T10-L2, T2-T12, TPA, Max Cobb, Max Cobb Apex), and desired postoperative PI-LL.
Johnson et al. (2023) (Johnson et al., 2023)	Spineview®, ENSAM, Laboratory of Biomechanics, Paris, France	Model 1: SVM for baseline predictive ability of the pertinent clinical data; Model 2: deep learning CNN (two Resnet18 models; one for lateral, and one for posterior-anterior radiograph inputs); Model 3: deep learning CNN on thoracic T1 MRIs.	70:20:20	133:38:20	Age, BMI, gender, comorbidities, previous fusion surgery.	Pelvic fixation, total instrumented levels, UIV and LIV level, coronal measurements (C7 plumb line, Major curve apex, Major curve Cobb angle, small curve apex deviation, small curve Cobb angle, T1 tilt, Thoracic curve apex deviation), sagittal measurements (C2 slope, cervical lordosis, cervicothoracic pelvic angle, L1-L4 angle, L1-S1, L1 pelvic angle, L4-S1 angle, LL, PI, PI-LL, PT, SS, C2-C7 cervical SVA, C7SVA, T1 spinopelvic inclination.
Lee et al. (2023) (Lee et al., 2023)	Surgimap v2.3.2.1 (Nemaris Inc., Methuen, MA)	SVM, RF, LDA, CART, KNN models	70:0:30	140:0:61	Age, BMI	Deformity type, SRS-curve, pattern, SRS-PI–LL modifier, SRS-global balance modifier, PI, at baseline, PJA at immediate postoperative state.
yu (2023) (Ryu et al., 2023)	NR	LR, DT, RF and GB models.	70:0:30	147:0:63	Age, Sex, surgical index level(s), height, weight, BMI, and history of spinal surgery.	Preop: PT, TK, T1 slope, C7SVA, SVA Postop: PT, TK, T1 slope, C7SVA, SVA, mean change of SVA
Tretiakov et al. (2023) (Tretiakov et al., 2023)	SpineView (ENSAM, Laboratory of Biomechanics)	Backstep conditional binary SL models	70:0:30	545:0:234	HRQOL, Short-Form 36-Item Health Survey, Oswestry Disability Index, and SRS-22 revised, mASD-FI	Schwab criteria, age-adjusted, GAP score, SAAS score PT, PI-LL, SVA

AI: Artificial Intelligence; ANN: Artificial Neural Network; ASD: Adult Spinal Deformity; BMD: Bone Mineral Density; BMI: Body Mass Index; CART: Classification and Regression Tree; CNN: Convolutional Neural Network; C7SVA: C7 Sagittal Vertical Axis; DT: Decision Tree; GAP: Global Alignment and Proportion; GB: Gradient Boosting Ensemble; LDA: Linear Discriminant Analysis; LIV: Lower Instrumented Vertebra; LL: Lumbar Lordosis; LR: Linear Regression; ML: Machine Learning; mASD-FI: ASD Frailty Index; MRI: Magnetic Resonance Imaging; NR: Not Reported; PI-LL: Pelvic Incidence Minus Lumbar Lordosis Mismatch; PT: Pelvic Tilt; RF: Random Forest; SAAS: Sagittal Age-Adjusted Score; SL: Supervised Learning; SRS: Scoliosis Research Society; SS: Sacral Slope; SVM: Support Vector Machine; TK: Thoracic Kyphosis; TPA: T1 Pelvic Angle; UIV: Upper Instrumented Vertebra; 3-CO: 3-Column Osteotomy.

**Table 5:** PJK/PJF/PJA definitions and calculations.

First Author and year	PJK definition	PJF definition	PJA calculation
Scheer et al. (2016) (Scheer et al., 2016)	Increase in the PJA by 20° or more compared with baseline and deterioration by at least 1 SRS-Schwab sagittal modifier grade from the 6 weeks post-operative time point of interest.	NR	Sagittal Cobb angle between the lower end plate of the UIV and the upper end plate of the UIV+2
Yagi et al. (2018) (Yagi et al., 2018)	NR	Increase ≥20° PJA, with concomitant deterioration of at least one Schwab-SRS sub-category grade compared to baseline immediately postoperative, or as any type of PJK requiring revision surgery	NR
Lafage et al. (2021) (Lafage et al., 2021)	Sagittal Cobb angle of UIV and UIV+2 > 10° and a postoperative change in UIV/UIV+2 > 10°.	Fracture of UIV or UIV+1, failure of UIV fixation, PJK of 15° of more, or need for extension of instrumentation within 6 months of surgery	NR
Johnson et al. (2023) (Johnson et al., 2023)	Sagittal Cobb angle of UIV and UIV+2 > 10° and a postoperative change in UIV/UIV+2 > 10°.	NR	NR
Lee et al. (2023) (Lee et al., 2023)	PJA of ≥20°, or an increase in PJA of ≥10° compared to the preoperative values.	NR	Sagittal Cobb angle between the lower end plate of the UIV and the upper end plate of the UIV+2
Ryu et al. (2023) (Ryu et al., 2023)	PJA between the UIV and vertebra level above the two vertebrae at the level of UIV (UIV + 2) > 10°.	Symptomatic PJK, with postoperative PJA >15°, associated with a possible requirement of revision such as in the case of fracture, soft-tissue failure, pullout of instrumentation at UIV, and/or sagittal subluxation.	NR
Tretiakov et al. (2023) (Tretiakov et al., 2023)	≥10° in sagittal Cobb angle between the inferior UIV endplate and the superior endplate of UIV + 2 vertebrae.	Subsequent revision surgery for PJK or a proximal junctional sagittal Cobb angle ≥15° with or without evidence of vertebral body fracture, implant fracture or displacement, or disruption of the osseo-ligamentous complex.	NR

NR: Not Reported; PJA: Proximal Junctional Angle; PJK: Proximal Junctional Kyphosis; PJF: Proximal Junctional Failure; SRS: Scoliosis Research Society; UIV: Upper Instrumented Vertebra.

### 3.5 Demographic, surgical, radiographical and outcome characteristics

Table 6, Table 7 summarize the demographic, surgical, radiographical, and outcome characteristics of the included studies. The studies included a mean of 311 ± 241 patients, with an average of 204 females (65.5 %). All studies provided baseline and final follow-up images, with some also including follow-up images at 6 weeks and 2 or 3 months (Scheer et al., 2016; Yagi et al., 2018). The average age of patients was 63.2 ± 3.7 years, the mean BMI was 26.1 ± 2.4 kg/m^2^, and the average number of levels fused per procedure was 9.82 ± 1.8. Of the included patients, 58.4 % underwent primary surgery, while 41.6 % had revision surgery. A total of 4 studies reported the preoperative SRS-Schwab Coronal Curve Type, while 3 studies reported the preoperative SRS-Schwab GB modifier.

**Table 6:** Demographic**,** surgical, radiographical and outcome characteristics.

First Author (year)	Sample Size	Follow-up	Age (years)	M/F (n, %)	BMI (kg/m^2^)	Primary/revision surgery (n)	Preop SRS-Schwab Coronal Curve Type (%)	Preop SRS-Schwab global balance modifier	Number of fused levels (n, SD)	UIV (n, %)	LIV (n, %)	Patients with 3-CO (n, %)	Type of implant
Scheer et al. (2016) (Scheer et al., 2016)	510	Baseline, 6 weeks, 3 months, 1 year, and 2-year follow-up	57.2 ± 13.9	114 (22.3 %)/396 (77.6 %)	27.3 ± 5.9	200:310	Type N: 42.5 % Type T: 4 % Type L: 32.3 % Type D: 21.2 %	PT modifier: 0 (29.2 %), + (35.1 %), ++ (35.7 %) GA modifier: 0 (33.8 %), + (21.8 %), ++ (44.4 %) PI-LL modifier: 0 (33.5 %), + (15.3 %), ++ (51.2 %)	11.8 ± 3.7	C-S: 1.4 % T1-T5: 48.6 % T6-T9: 12 % T10-L3: 38 %	T11-L2: 31 (6.1 %), L3-L5: 80 (15.7 %), Sacro-iliac: 399 (78.2 %)	289 (56.7 %)	Hooks and screws
Yagi et al. (2018) (Yagi et al., 2018)	145	Baseline, 6 weeks and 2-year follow-up	63.9 ± 9.4	5 (4 %)/107 (96 %)	21.9 ± 3.9	101:11	Type N: 28 % Type T: 8 % Type L: 46 % Type D: 18 %	NR	9.9 ± 2.3	NR	Pelvis: 62 (55 %), L5 or above: 50 (45 %9	19 (17 %)	Screws
Lafage et al. (2021) (Lafage et al., 2021)	143	Baseline and 1-year	63.3 ± 10.6	26 (18.2 %)/117 (81.8 %)	27.1 ± 5.6	NR	Type N: 35 % Type T: 0 % Type L: 33.6 % Type D: 29 %	PT modifier: + or ++ (68.6 %); GA modifier: + or ++ (74.9 %); PI-LL modifier: + or ++ (74.2 %)	10.2 ± 3.5	T2: 8 %, T3: 12 %, T4: 11 %, T5: 4 %, T6: 1 %, T7: 2 %, T8:2 %, T9:8 %, T10: 29 %, T11:18 %, T12: 7 %	NR	36 (25.2 %)	NR
Johnson et al. (2023) (Johnson et al., 2023)	191	Baseline and 2-year	63.1 ± 18.4	45 (23.6 %)/146 (76.4 %)	28.8 ± 7.0	135:56	NR	NR	10.6 ± 3.0	T1: 0.5 %, T2: 4.2 %, T3: 7.9 %, T4: 14.1 %, T5: 7.9 %, T6: 2.6 %, T7: 3.1 %, T8: 9.4 %, T9: 9,9 %, T10: 23 %, T11: 7.9 %, T12: 5.8 %, T12: 5.8 %, L1: 0.5 %, L2: 3.1 %	NR	NR	NR
Lee et al. (2023) (Lee et al., 2023)	201	Baseline and 1-year	67.16 ± 9.08	43 (21.4 %)/158 (78.6 %)	24.71 ± 3.94	145: 56	Type T: 1.5 % Type L: 95 % Type D: 31.8 % Primary sagittal: 51.2 %	PT modifier: 0 (15.1 %), + (30.3 %), ++ (51.1 %)/PI-LL modifier: <10° (11.2 %), 10–20° (30.3 %), >20° (68.4 %)/GA modifier: 0 (24.3 %), + (28.3 %), ++ (38.8 %).	7.14 ± 2.97	NR	Ileum screw: 51 (33.6 %), S2-alar screw: 4 (2.6 %), S2-alar-ileum screw: 8 (5.3 %)	III: 68 (34 %)/IV: 6 (3 %)/V: 4 (2 %)	NR
Ryu et al. (2023) (Ryu et al., 2023)	210	Baseline and 2-year	URO: 66.9 ± 6.6/No URO: 68.9 ± 8.7	31 (14.7 %)/179 (85.3 %)	URO: 25.0 ± 3.7/No URO: 24.3 ± 3.4	152: 58	NR	NR	URO: 7.3 ± 2.2/No URO: 7.8 ± 2.1	NR	NR	URO: 23.6 ± 20.3/No URO: 26.2 ± 21.4	NR
Tretiakov et al. (2023) (Tretiakov et al., 2023)	779	Baseline and 2-year	59.87 ± 14.24	172 (22 %)/607 (78 %)	27.78 ± 6.02	NR	NR	NR	11.6 ± 4.1	NR	NR	522 (67 %)	Hook and screws

BMI: Body Mass Index; C-S: Cervical Spine; GA: Global Balance; LIV: Lower Instrumented Vertebra; PI-LL: Pelvic Incidence Minus Lumbar Lordosis Mismatch; PT: Pelvic Tilt; NR: Not Reported; SRS: Scoliosis Research Society; UIV: Upper Instrumented Vertebra; URO: Unplanned Reoperation; 3-CO: 3-Column Osteotomy.

**Table 7:** Cases/controls and radiographical characteristics.

First Author (year)	PJK/PJF/controls (n, %)	Radiographical characteristics highlighted in the studies
Scheer et al. (2016) (Scheer et al., 2016)	PJK/PJF group: 139 (27.2 %)/None group: 371 (72.8 %)	The PJK/PJF group exhibited significantly higher mean baseline PT, PI-LL, and SVA compared to the None group. Both groups had a similar baseline mean TK.
Yagi et al. (2018) (Yagi et al., 2018)	PJF group: 22 (20 %)/None group: 90 (80 %)	LIV at the pelvis, SRS-Schwab type N or L, severe PT, low UIV, and high baseline PI-LL mismatch were significant risk factors for PJF.
Lafage et al. (2021) (Lafage et al., 2021)	PJK group: 42 (29.4 %)/None group: 101 (70.6 %)	Patients showed significant changes in lumbar alignment (ΔPI-LL: 21° ± 16, p < 0.001). The UIV was in the UT for 35 % and in the LT for 65 %.
Johnson et al. (2023) (Johnson et al., 2023)	PJK group: 89 (46.6 %)/None group: 102 (53.4 %)	NR
Lee et al. (2023) (Lee et al., 2023)	PJK group: 49 (24.4 %)/None group: 152 (75.6 %)	After deformity correction, 42 % of patients achieved the ideal age-adjusted PI-LL, 44.5 % were under corrected, and 13.4 % were overcorrected.
Ryu et al. (2023) (Ryu et al., 2023)	PJF group: 83 (39.5 %)/None group: 127 (60.5 %)	An increase in postoperative SVA is positively correlated with a higher risk of PJF.
Tretiakov et al. (2023) (Tretiakov et al., 2023)	PJK/PJF group: 472 (60.6 %)/None group: 307 (39.4 %)	Patients who developed PJK or PJF had greater PT and TPA offsets, were more likely to be under corrected according to SAAS scoring, and had a UIV of T10 or lower, or LIV at the sacrum, all of which were significant risk factors for PJK or PJF.

LIV: Lower Instrumented Vertebra; NR: Not Reported; PJK: Proximal Junctional Kyphosis; PJF: Proximal Junctional Failure; PI-LL: Pelvic Incidence Minus Lumbar Lordosis Mismatch; PT: Pelvic Tilt; SAAS: Sagittal Age-Adjusted Score; SRS: Scoliosis Research Society; SVA: Sagittal Vertical Axis; TK: Thoracic Kyphosis; TPA: T1 Pelvic Angle; UIV: Upper Instrumented Vertebra; UT: Upper Thoracic.

A total of 896 patients developed either PJK or PJF at the last follow-up (41.1 %). Scheer et al. found that the PJK/PJF group had significantly higher mean baseline PT, PI-LL, and SVA than the None group (Scheer et al., 2016). Yagi et al. identified LIV at the pelvis, Schwab-SRS type N or L, severe PT, low UIV, and high baseline PI-LL mismatch as significant risk factors for PJF (Yagi et al., 2018). Tretiakov et al. concluded that patients with greater PT and TPA offsets, those more likely to be under-corrected according to Sagittal Age-adjusted Score (SAAS) scoring, and those with a UIV of T10 or lower or LIV at the sacrum, were more prone to PJK or PJF in the postoperative period (Tretiakov et al., 2023). Lee et al. found that after deformity correction, 42 % of patients achieved the ideal age-adjusted PI-LL, 44.5 % were under-corrected, and 13.4 % were overcorrected (Lee et al., 2023).

### 3.6 ML-models’ results

Table 8 presents a summary of the results of the ML models, along with their characteristics, including sensitivity, specificity, accuracy, precision, recall, AUC/AUROC, and AUPRC, as well as the findings and conclusions of the included studies.

**Table 8:** AI outcomes and results.

First Author and year	Sensitivity/Specificity	Accuracy	Precision	Recall	AUC/AUROC/AUPRC	Results	Conclusions
Scheer et al. (2016) (Scheer et al., 2016)	NR	86.3 %	NR	NR	0.89	The 7 most significant predictors (importance ≥0.95) were: age, LIV, preoperative SVA, UIV implant type, UIV, preoperative PT, and preoperative PI-LL.	An effective model was developed with 86 % accuracy and a 0.89 AUC to predict either PJF or clinically relevant PJK.
Yagi et al. (2018) (Yagi et al., 2018)	NR	Without BMD: 80.4 % (training)/75.7 % (testing)/With BMD: 98.1 % (training)/100 % (testing)	NR	NR	1.0 (testing with BMD)	The 9 most important predictors were: PT, BMD, LIV level (pelvis), UIV level, PSO application, global alignment (C7SVA), BMI, PI-LL, and age.	A robust model was created to predict PJF, incorporating BMD as a factor. This model could help guide physicians in identifying patients at high risk of developing PJF during the perioperative period.
Lafage et al. (2021) (Lafage et al., 2021)	NR	81 %	87.5 %	87.5 %	0.829	Post-operative comparison between the UT and LT groups revealed greater thoracic kyphosis and a larger residual coronal Cobb angle in the UT group, with no significant differences in other parameters or PJK rate.	An artificial neural network effectively replicated the decision-making process of two lead surgeons in choosing the UIV for ASD correction.
Johnson et al. (2023) (Johnson et al., 2023)	Model 1: SE: 57.2 %; SP: 56.3 % Model 2: SE: 68.2 %; SP: 58.3 % Model 3: SE: 73.1 %; SP: 79.5 %.	NR	NR	NR	NR	An attention map revealed that soft tissue features were the primary factors in all true positive PJK predictions.	Using raw MRIs in an AI model enhanced PJK prediction accuracy over scoliosis radiographs and traditional measurements, suggesting that soft tissue degeneration and muscle atrophy are key factors in predicting PJK.
Lee et al. (2023) (Lee et al., 2023)	RF: SE: 0.57; SP:0.94, SVM: SE: 0.29; SP: 1.0; LDA: SE: 0.14; SP: 0.97, CART: SE: 0.36; SP: 0.79, KNN: SE: 0.07; SP: 0.85.	RF: 0.83, SVM: 0.79, LDA: 0.72, CART: 0.66, KNN: 0.62.	NR	NR	RF: 0.76, SVM: 0.64, LDA: 0.56, CART: 0.57, KNN: 0.46.	The RF model achieved the highest accuracy at 83 %, with an area under the receiver operating characteristics curve of 0.76.	An online calculator, founded on the random forest model, has been developed to gauge the risk of PJK following ASD surgery. This may be a useful clinical tool for surgeons, allowing them to better predict PJK probabilities and refine subsequent therapeutic strategies.
Ryu et al. (2023) (Ryu et al., 2023)	NR	LR: 65.6 %; DT: 61.7 %; RF: 72.5 %; GB: 71.3 %.	NR	NR	LR: 0.66/0.68, DT: 0.59/0.65, RF: 0.73/0.82, GB: 0.68/0.81	Machine learning-identified risk factors, including URO, PJF, and postoperative SVA, were significant in the Kaplan-Meier survival analysis.	Machine learning and game theory identified postoperative SVA, PJF, and their interactions as major risk factors for URO after ASD surgery.
Tretiakov et al. (2023) (Tretiakov et al., 2023)	NR	NR	NR	NR	0.923	The six key predictors of PJK/PJF were age ≥74, high baseline SAAS T1 pelvic angle modifier, high PT modifier, a fusion of >10 levels, no prophylaxis use, and a 6-week SAAS PI-LL modifier >1.	This study presents a validated model that predicts clinically significant PJK and PJF, aiding in patient selection, and intraoperative decisions, and reducing postoperative complications in ASD surgery.

AUC: Area Under The Curve; AUPRC: Area Under The Precision-Recall Curve; AUROC: Area Under the Receiver Operating Characteristics; BMD: Body Mass Density; BMI: Body Mass index; CART: Classification and regression tree; CNN: convolutional neural network; DT: Decision Tree; GB: Gradient boosting ensemble; KNN: K-nearest neighbors; LDA: linear discriminant analysis; LIV: Lower Instrumented Vertebra; LR: Linear Regression; LT: Lower Thoracic; MRIs: Magnetic Resonance Images; NR: Not Reported; PI-LL: pelvic incidence minus lumbar lordosis mismatch; PJK: Proximal Junctional Kyphosis, PJF: Proximal Junctional Failure; PSO: Pedicle Subtraction Osteotomy; PT: pelvic tilt; SAAS: Sagittal Age-adjusted Score; SE: Sensitivity; SL: Supervised Learning; SP: Specificity; SVA: sagittal vertical axis; SVM: Support Vector Machine; TK: thoracic kyphosis; TPA: T1 pelvic angle; UIV: Upper Instrumented Vertebra; URO: Unplanned Reoperation: UT: Upper Thoracic.

#### 3.6.1 Assessment of key predictors of PJK/PJF Occurrence

Scheer et al.'s C5.0 algorithm decision tree predicted the onset of PJK/PJF with an overall accuracy of 86.3 % and an AUC of 0.89. The seven most significant predictors identified were age, LIV, preoperative SVA, UIV implant type, UIV, preoperative PT, and preoperative PI-LL (Scheer et al., 2016). Yagi et al. trained two separate decision-making trees using the Tobuilda and C5.0 algorithms, one incorporating Bone Mineral Density (BMD) values and one without, finding that the inclusion of BMD significantly enhanced prediction accuracy, achieving an AUC of 1.0 when BMD was included. The nine most significant predictors for PJK were PT, BMD, LIV level (pelvis), UIV level, pedicle subtraction osteotomy (PSO) application, global alignment (C7-SVA), BMI, PI-LL, and age (Yagi et al., 2018). Tretiakov et al. achieved an AUC of 0.923 using a backstep conditional binary Supervised Learning (SL) model, identifying six key predictors of PJK/PJF: age ≥74, high baseline SAAS TPA modifier, high PT modifier, fusion of >10 levels, no prophylaxis use, and a 6-week SAAS PI-LL modifier >1 (Tretiakov et al., 2023). In conclusion, Lee et al. used five different ML-models and found that the Random Forest (RF) model had the highest accuracy (83 %) and AUROC (0.76) (Lee et al., 2023).

#### 3.6.2 Preventive measures for PJK development

Tretiakov et al. found that prophylactic measures like cementing, hooks, tethers, and hybrid methods could help prevent PJK and PJF, with the SAAS scoring system being a strong predictor of junctional degeneration (Tretiakov et al., 2023). Yagi et al. emphasized the importance of BMD in predicting PJK/PJF and recommended low BMD as a key factor, advising teriparatide treatment and UIV+1 tethering as preventive strategies (Yagi et al., 2018).

#### 3.6.3 Soft tissue characteristics’ impact in PJK

Johnson et al. developed three distinct machine learning models to predict PJK following ASD surgery. Their final model (Model 3), which utilized raw thoracic T1 magnetic resonance images (MRIs), achieved higher sensitivity (73.1 %) and specificity (79.5 %) compared to earlier models. The study concluded that soft tissue features were the primary factors in all accurate positive radiographic PJK predictions (Johnson et al., 2023).

#### 3.6.4 Replication of surgeon decision-making in UIV selection

Lafage et al.'s analysis focused on developing an ANN to replicate surgeon decision-making in UIV selection, using preoperative data and alignment objectives, and to assess the risk of PJK postoperatively. Their results showed an accuracy of 81 %, precision and recall of 87.5 %, and an overall AUC of 0.829, revealing a larger residual coronal Cobb angle and greater TK in the Upper Thoracic (UT) group, though there were no significant differences in PJK rates (Lafage et al., 2021).

#### 3.6.5 Identification of major risk factors for Unplanned Reoperations (UROs)

Ryu et al. developed four distinct ML models to identify the key risk factors, including PJK and PJF, for UROs following corrective surgery for ASD. Their analysis found that the RF model demonstrated the highest accuracy (72.5 %) and AUROC/AUPRC (0.73/0.82), with postoperative SVA, PJF, and their interactions identified as key risk factors for UROs following ASD surgery (Ryu et al., 2023).

## 4 Discussion

This systematic review analyzed data from 2179 patients across seven studies evaluating AI models for predicting and preventing PJK/PJF following ASD surgery. The findings highlight the potential of ML algorithms in managing these complex cases, demonstrating that various models can be effectively and flexibly utilized to predict ASP development in many surgically treated ASD patients.

### 4.1 Strengths, limitations, and applications of AI predictive models in ASD surgery

Over the past decade, AI has revolutionized medicine by improving diagnostic imaging, disease prediction, and patient management, leading to better clinical outcomes (Kaul et al., 2020; Rajpurkar et al., 2022; Agnieszka and Hanna, 2023). Notably, ML models have shown promise in predicting postoperative outcomes and reducing complications, offering improved accuracy by identifying data relationships and minimizing bias compared to traditional methods (Tragaris et al., 2023; Joshi et al., 2021a). The training of these models involves testing with common splits of 80:20 or 70:30 and iterative cross-validation on separate datasets to optimize predictive performance (Joshi et al., 2021a). As a result, ML models offer significantly more powerful predictive capabilities than statistical models, but their complexity often makes them more challenging to interpret (Cina and Galbusera, 2024). Indeed, its integration presents challenges such as automation bias, ethical concerns regarding data privacy, algorithm transparency, and decision-making biases, which require careful attention (Lyell and Coiera, 2017; Aldosari and Alanazi, 2024). To maximize AI's benefits while minimizing risks, rigorous validation, regulatory oversight, and collaboration with clinical expertise are necessary (Jeyaraman et al., 2023).

AI models have become increasingly prevalent in spinal surgery, assisting clinicians in early imaging assessment, automated analysis of radiographic parameters, treatment planning, and prognostic predictions (Joshi et al., 2021b; Bajwa et al., 2024). Hence, AI-driven algorithms have been developed to enhance surgical planning and optimize patient-specific strategies in ASD, a complex condition that requires meticulous treatment planning and could greatly benefit from ML predictive models (Joshi et al., 2019, 2021b; Dalton et al., 2023; Lopez et al., 2021). A range of predictive tools have already been employed in ASD management, extending from forecasting the length of hospital stay and the need for blood transfusions to predicting perioperative or postoperative complications such as pseudoarthrosis, malalignments and ASP (Durand et al., 2018; Safaee et al., 2018; Scheer et al., 2017, 2018; Passias et al., 2016).

### 4.2 Key predictors and preventive measures for PJK/PJF occurrence

Over the years, numerous radiographical classification methods have been applied to predict and prevent ASP (Dagdia et al., 2019). Indeed, radiological parameters may play a key role in assessing the risk of complications following ASD surgery, with tools such as the SRS-Schwab Classification, Age Adjusted Sagittal Alignment, GAP score, and Roussouly Classification being commonly used (Terran et al., 2013; Lafage et al., 2017; Yilgor et al., 2017; Berthonnaud et al., 2005). However, no single system can reliably predict such complications, suggesting the need to adopt of a holistic approach that combines various patient-specific factors and predictive models to achieve optimal outcomes (Yagi et al., 2023). Therefore, preoperative planning must include careful considerations, such as assessing the patient's BMD and modifying the surgical technique (Yagi et al., 2016; Kendler et al., 2018; Ohtori et al., 2013; Duan et al., 2020). These adjustments may involve avoiding overcorrection, limiting fusion levels, employing cement-augmented pedicle screws at the UIV and UIV+1, adding ligament augmentation at the UIV levels, and contouring the terminal rod (Yagi et al., 2023). Two studies in this review explored the impact of prophylactic surgical techniques on PJK/PJF, with Tretiakov et al. suggesting cementing and tethers as preventive measures and Yagi et al. highlighting low BMD as a key factor, recommending teriparatide and UIV+1 tethering for prevention (Tretiakov et al., 2023; Yagi et al., 2018).

### 4.3 The impact of soft tissue characteristics on PJK

ML models are trained using MRI images in various medical fields (Khan et al., 2023; Reig et al., 2020; Altabella et al., 2022). In spinal surgery, these images are frequently used to assess lumbar intervertebral disc degeneration, muscle composition, and their role in disease progression (Mallio et al., 2024). For example, Tian et al. developed an MRI-based predictive model for PJK/PJF, combining bone and paraspinal muscle quality metrics, and found that paraspinal muscle and vertebral bone quality are more influential than radiographic alignment in predicting PJK/PJF in ASD correction patients (Tian et al., 2024). In the included study by Johnson et al., two predictive algorithms were developed: the first (Model 1) using only demographic, radiographic variables, and intended fusion levels, and the second (Model 2) incorporating raw scoliosis images. A third model (Model 3) was then created by adding preoperative thoracic T1 MRI images to the variables from Model 1. This model demonstrated a significant increase in sensitivity and specificity in predicting radiographic PJK risk, concluding that soft tissue information and its degeneration at the T1 level may play a crucial role in forecasting postoperative ASP (Johnson et al., 2023).

### 4.4 The influence of UIV selection on the development of PJK

The selection of the UIV in ASD correction is considered a crucial aspect. UIV in the UT spine may provide more stable correction and lower rates of PJK and PJF, while a UIV in the LT spine may offer benefits such as reduced operative time, blood loss, costs, and risk of soft tissue failure, especially in high-BMI patients (Virk et al., 2021). Kumar et al. recently proposed an algorithm for selecting the UIV in long-segment fusions, concluding that the choice should be made after considering various patient characteristics, including preoperative alignment, comorbidities, as well as surgeon-specific and facility-specific factors (Kumar et al., 2024). Nevertheless, a consensus on the selection of the UIV in ASD surgery has yet to be reached. The study by Lafage et al. in this review aimed to create an algorithm that replicates the decision-making process of experienced surgeons in selecting the UIV, incorporating demographic data, preoperative coronal and sagittal alignment, postoperative pelvic incidence-lumbar lordosis mismatch, and postoperative alignment goals (Lafage et al., 2021). They found that, at one year, the revision rate was 11.9 %, and the rate of radiographic PJK was 29.4 %, with no significant difference between the UT and LT groups. Thus, predictive algorithms offer considerable potential in assisting with the accurate selection of the UIV, thereby opening the door for future research focused on validating these algorithms for UIV prediction and PJK/PJF prevention in the treatment of ASD (Lafage et al., 2021).

### 4.5 Selecting the correct AI predictive model

The selection of the appropriate AI model and the rationale behind its use remains an ongoing subject of debate. Lee et al. and Ryu et al. demonstrated that models like RF can achieve the highest accuracy and AUC in predicting the development of PJK and identifying key factors influencing UROs (Lee et al., 2023; Ryu et al., 2023). However, Tretiakov et al. employed a SL predictive analysis to improve the transparency of the factors being studied, emphasizing that RF models, while powerful, can lead to an overestimation of target outcomes due to increased out-of-bag error in binary classification tasks (Tretiakov et al., 2023). This highlights the importance of carefully considering model limitations and the specific context in which they are applied (Janitza and Hornung, 2018). For this reason, further comprehensive studies are required to validate the superiority of one model over another.

### 4.6 Limitations

The present review has several limitations that must be acknowledged. Due to their absence in the literature, no RCTs or prospective studies were included. Additionally, there is a significant lack of consistency in the predictive algorithms, training variables, definitions of PJK/PJF, and intervention outcomes across the included studies. In conclusion, the algorithms were trained on a relatively small number of patients, with some studies including as few as 143 patients, which limits the ability to draw definitive conclusions on the subject.

For these reasons, there is an urgent need for prospective multicenter RCTs with larger sample sizes that incorporate the parameters outlined in the following study, mainly including a precise assessment of patients' BMD values, utilizing all radiographic variables (SRS-Schwab Classification, Age Adjusted Sagittal Alignment, GAP score, Roussouly Classification, and SAAS scoring system), employing a standardized definition of PJK/PJF, thoroughly assessing the impact of prophylactic measures and UIV selection, and integrating the algorithm with preoperative MRI images are essential steps to further validate the findings of this review.

Nevertheless, the current review highlights that studies utilizing ML predictive algorithms for evaluating postoperative PJK/PJF in ASD patients show potential, offering valuable insights into improving outcomes and enhancing surgical planning and patient management.

## 5 Conclusion

The ability to predict and prevent the development of ASP with certainty in the complex surgical context of ASD patients could represent a pivotal breakthrough. The present analysis provides convincing evidence of the considerable potential of ML algorithms in the prediction and prevention of PJK/PJF. Future research should focus on prospective multicenter RCTs with larger sample sizes, employing a standardized definition of PJK/PJF, careful evaluation of prophylactic measures and UIV selection, accurate assessments of patients' BMD values, a comprehensive range of radiographic variables and scoring systems, and the integration of preoperative MRI images, to further validate the findings of this review.

## CRediT authorship contribution statement

**Paolo Brigato:** Conceptualization, Data curation, Formal analysis, Methodology, Software, Writing – original draft. **Gianluca Vadalà:** Formal analysis, Writing – review & editing. **Sergio De Salvatore:** Conceptualization, Data curation, Methodology, Writing – original draft. **Leonardo Oggiano:** Data curation, Methodology. **Giuseppe Francesco Papalia:** Software. **Fabrizio Russo:** Visualization, Writing – review & editing. **Rocco Papalia:** Supervision, Visualization. **Pier Francesco Costici:** Supervision. **Vincenzo Denaro:** Supervision, Validation, Visualization, All authors have read and agreed to the published version of the manuscript.

## Ethical approval

Not required.

## Availability of data and materials

Not required.

## Funding

This work was supported by the 10.13039/501100000780European Union – Next Generation EU – NRRP M6C2 – Investment 2.1 Enhancement and strengthening of biomedical research in the 10.13039/100030827NHS [PNRR-MAD-2022-12376692_VADALA’ - CUP F83C22002470001].

## Declaration of competing interest

The authors declare that they have no known competing financial interests or personal relationships that could have appeared to influence the work reported in this paper.

## Abbreviations:

AUC: Area Under the Curve
AUROC: Area Under the Receiver Operating Characteristics
AUPRC: Area Under The Precision-Recall Curve
ANNs: Artificial Neural Networks
ASP: Proximal Adjacent Segment Pathology
AI: Artificial Intelligence
BMI: Body Mass Index
BMD: Bone Mineral Density
CNNs: Convolutional Neural Networks
DL: Deep Learning
LOE: Level of Evidence
LIV: Lower Instrumented Vertebra
PFA: Proximal Junctional Angle
PJK: Proximal Junctional Kyphosis
PJF: Proximal Junctional Failure
PI: Pelvic Incidence
PI-LL: Pelvic Incidence minus LL mismatch
PT: Pelvic Tilt
RC: Retrospective Cohort studies
RF: Random Forest
RCTs: Randomized Controlled Trials
SAAS: Sagittal Age-adjusted Score
SRS: Scoliosis Research Society
SL: Supervised Learning
SVA: Sagittal Vertical Axis
TK: Thoracic Kyphosis
UIV: Uppermost Instrumented Vertebra
UT: Upper Thoracic
3-CO: 3-Column Osteotomies
URO: Unplanned Reoperations
